# Bicarinalin Enhances the Antibacterial Activity of Levofloxacin and Clarithromycin Against *Helicobacter pylori*

**DOI:** 10.3390/antibiotics14101003

**Published:** 2025-10-10

**Authors:** Iman Saleh, Pınar Küce Çevik

**Affiliations:** Department of Molecular Biology and Genetics, Faculty of Natural and Applied Sciences, Harran University, Sanliurfa 63050, Turkey

**Keywords:** *H. pylori*, Bicarinalin, AMP, SEM, antibacterial activity, MIC

## Abstract

**Background/Objectives:** *Helicobacter pylori* (*H. pylori*) is a Gram-negative bacterium that colonizes the human stomach and causes various gastrointestinal diseases. Although antibiotic therapy is the most effective method for its eradication, the increasing prevalence of antibiotic resistance has made treatment increasingly challenging in recent years. In this study, the antimicrobial activity, synergistic effects with antibiotics, and mechanisms of action of Bicarinalin, an antimicrobial peptide (AMP) derived from the venom of *Tetramorium bicarinatum*, were investigated against *H. pylori*. **Methods:** To determine the antibacterial activity of Bicarinalin, a well diffusion assay was performed, yielding an inhibition zone of 18.3 mm at a concentration of 32 µg/mL for ATCC strain. MIC_99_ values were determined by microdilution tests as 4.8 μg/mL for the reference strain. The enhancement of the antimicrobial potential of levofloxacin and clarithromycin when administered together with Bicarinalin has been demonstrated using the well diffusion method. **Results:** Inhibition zones increased from 14.2 mm to 20 mm for levofloxacin and from 7.3 mm to 16 mm for clarithromycin. This study is the first to identify DNA and protein leakage caused by Bicarinalin in *H. pylori*. Intracellular protein and DNA leakage were measured, with protein and DNA levels released into the extracellular environment determined as 33.25% and 55.10%, respectively, following Bicarinalin treatment. Furthermore, to investigate its effect on membrane damage, scanning electron microscopy (SEM) was performed, revealing disrupted cell membrane structures, penetration between cells, and severe deterioration of morphological integrity. **Conclusions:** This study has demonstrated for the first time that, when administered concomitantly, Bicarinalin enhances the antimicrobial activities of levofloxacin and clarithromycin. This highlights its potential as an adjunctive treatment for *H. pylori* alongside existing drugs.

## 1. Introduction

*Helicobacter pylori* (*H. pylori*) is a Gram-negative bacterium [1] and one of the most common pathogens, with an estimated prevalence of infection in approximately half of the global population [2]. Infection with *H. pylori* results in gastrointestinal inflammation and other conditions such as peptic ulcer, gastric cancer, mucosa-associated lymphoid tissue lymphoma, and several other disorders [3]. There is currently no treatment that can fully eradicate *H. pylori* in all cases because the bacterium possesses acid resistance mechanisms and colonization factors that allow it to persist in the gastric environment [4]. It develops a cover of acid neutralizing substances around itself through the urease enzyme [5]. In recent years, the global prevalence of *H. pylori* infection has resulted in the widespread use of antibiotics, which has in turn led to the emergence of multidrug resistance [6,7]. In clinical practice, *H. pylori* is typically managed with combined drug therapies and proton pump inhibitors. However, the rapid spread of drug resistance has made it imperative to investigate alternative strategies for managing this infection [6,8]. Hence, the complete eradication of *H. pylori* and finding alternative therapy holds great importance in the treatment and prevention of gastrointestinal diseases [9]. There is an immediate demand for the development of novel antibiotics or to improve the efficacy of antibiotics to guarantee the effectiveness of treatments against infections that are resistant to common antibiotics [10].

Antimicrobial peptides (AMPs) are recognized as relatively short amphipathic peptides composed of approximately 10 to 100 amino acids, generally carrying a positive net charge and often adopting an alpha-helical structure [11]. AMPs, which are commonly found in humans, animals, plants, arthropods, and microorganisms, play a crucial role in innate immunity by acting as the first-line defense against invading pathogens and have a broad-spectrum effect against bacteria, fungi, and viruses [12,13]. AMPs emerge as promising alternatives to traditional antibiotics, particularly for treating multidrug-resistant microbes, viral infections, cancer, and inflammatory disorders [14]. Despite challenges, researchers are making strides in unlocking their full therapeutic potential [15,16]. Currently, approximately 50 AMP-based drugs are in the clinical trial phase, of which 14 have reached Phase III and only 11 have made it to market [17]. Recent studies show that AMPs can have synergistic effects with conventional antibiotics, offering the potential to combat superbugs, reduce the development of drug resistance, and enhance antibiotic efficacy [18]. Investigating the efficacy of AMPs against various pathogens, either singly or synergistically, is very important to develop new therapeutic approaches.

Bicarinalin is a cationic, C-terminus amidated, α-helical linear, novel AMP which is isolated from the *Tetramorium bicarinatum myrmicine* ant venom [19]. It contains twenty amino acid residues with the sequence of (KIKIPWGKVKDFLVGGMKAV) and its molecular weight is 2213.78 g/mol [20]. The peptide has a broad-spectrum antimicrobial effect on a variety of bacteria, parasites, and fungi within a minimum inhibitory concentration of 0.45 μmol/L (*Staphylococcus xylosus*) and 97.5 μmol/L (*Geotrichum candidum*). In addition, at low concentrations from 0.066 to 8.5 μmol/mL, it exhibits no hemolytic and cytotoxic effect to human lymphocytes. Bicarinalin is stable against proteases and can survive in the body for up to 15 h. Furthermore, its positive pharmacokinetic profile demonstrates its potency for use in antimicrobial chemotherapy [21]. This novel and authentic peptide possess no sequence similarities with previously cataloged molecules in existing databases [19]. Bicarinalin has the potential to be a candidate to develop a new antimicrobial drug [22]. It is hypothesized that Bicarinalin possesses an antibacterial mechanism of action analogous to that observed in other AMPs. The inhibition of bacterial growth is achieved through the permeabilization of bacterial membranes, resulting in the leakage of cellular contents into the external environment. In addition to showing antimicrobial activity against many pathogens, it is reported to prevent the attachment and settlement of pathogens in tissues [23].

Our study was aimed at demonstrating the potential of Bicarinalin as an agent to overcome *H. pylori*. There is no study, to date, that has determined the synergistic activity of Bicarinalin or shown the direct effect of the AMP on the membrane, DNA, and protein of *H. pylori.* Hence, another objective of our study was to assess the structural damage induced by Bicarinalin on the bacterial membrane using scanning electron microscopy (SEM) and determine the extent of intracellular content leakage—namely DNA and proteins—through established colorimetric assays.

## 2. Results

### 2.1. Inhibition Zone Values (Well Diffusion)

Well diffusion method of Bicarinalin with different concentrations on MHA is presented in (Table 1). The inhibition zones indicate the lowest concentration where Bicarinalin completely inhibited visible growth of *H. pylori* ATCC strain.

The inhibition zone diameters of Bicarinalin against commercial *H. pylori* strains were evaluated using the well diffusion method. These were observed to increase significantly in parallel with increasing concentration. Zone diameter of 18.3 mm was obtained in ATCC strain at a concentration of 32 µg/mL. At low concentrations (4–8 µg/mL), the zone diameters were limited to 7–8.7 mm. At a medium concentration of 16 µg/mL, the zone diameter increased to approximately 9.7 mm. These results clearly demonstrate that Bicarinalin exhibits a dose-dependent inhibitory effect on *H. pylori*.

### 2.2. Data of Microdilution Assay

**Figure 1:**
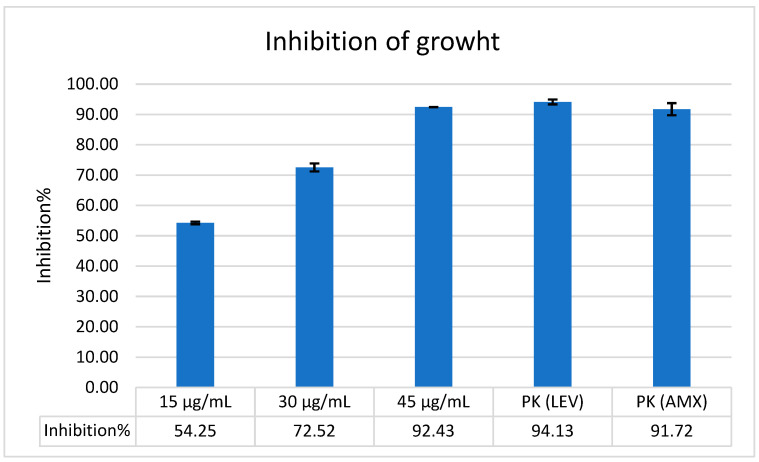
The chart graphic of growth inhibition percentage by Bicarinalin against *H. pylori* ATCC strain attained from the microdilution assay using the formula. The rate of growth inhibition increases when the concentration of Bicarinalin increases.

Microbroth dilution assay was employed to determine the minimum inhibitory concentration (MIC). Levofloxacin (LEV) and amoxicillin (AMX) served as positive controls. The mean optical density (OD) values, calculated from triplicate measurements, were analyzed using the specified formula and summarized in (Figure 1). This figure also includes corresponding growth rates and inhibition percentages for *H. pylori* ATCC strain. The MIC_99_ value—defined as the concentration required to inhibit 99% of bacterial growth—was determined as 4.8 μg/mL. Statistical evaluation of OD values was conducted using one-way ANOVA followed by Turkey’s HSD test. The results demonstrated a dose-dependent enhancement of Bicarinalin’s antimicrobial activity. A statistically significant difference was observed at the highest concentration tested, at which point Bicarinalin exhibited greater efficacy against the bacteria. Notably, at concentrations of 15 μg/mL and 30 μg/mL, Bicarinalin demonstrated stronger antibacterial activity against *H. pylori* than the reference antibiotics LEV and AMX.

### 2.3. The Antimicrobial Activity of Bicarinalin and Its Combination with Antibiotics

**Figure 2:**
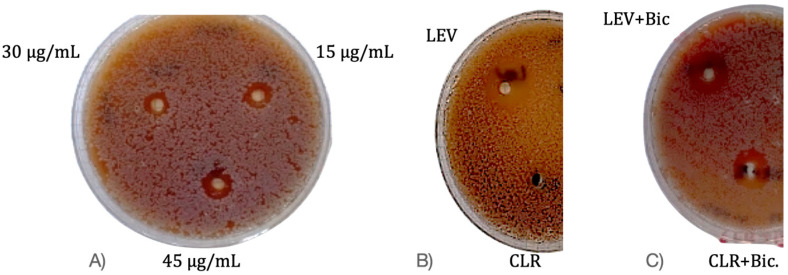
Synergistic activity of Bicarinalin. (**A**) The antibiotics (LEV, CLR, and TOB) on MHA against *H. pylori.* (**B**) Bicarinalin on MHA against *H. pylori.* (**C**) Bicarinalin combined with LEV and on MHA against *H. pylori*.

The efficacy of Bicarinalin against the *H. pylori* ATCC strain is shown in Figure 2 and Table 2. Figure 2A shows the inhibition zones obtained with Bicarinalin alone; the most pronounced effect was observed at 45 μg/mL. Figure 2B shows the inhibition zones produced by antibiotics alone; here, the bacteria exhibited the highest sensitivity to LEV. Figure 2C shows the inhibition zones resulting from the combination of Bicarinalin and antibiotics; here, the bacteria appear most sensitive to the combination of Bicarinalin and LEV. Although these results suggest that Bicarinalin has a potential to enhance the effectiveness when combined with certain antibiotics, they are insufficient to conclusively prove synergy. Further studies are needed in order to confirm whether the observed effect is real synergy or merely an enhancing activity.

### 2.4. Intracellular Protein and DNA Leakage Values

**Figure 3:**
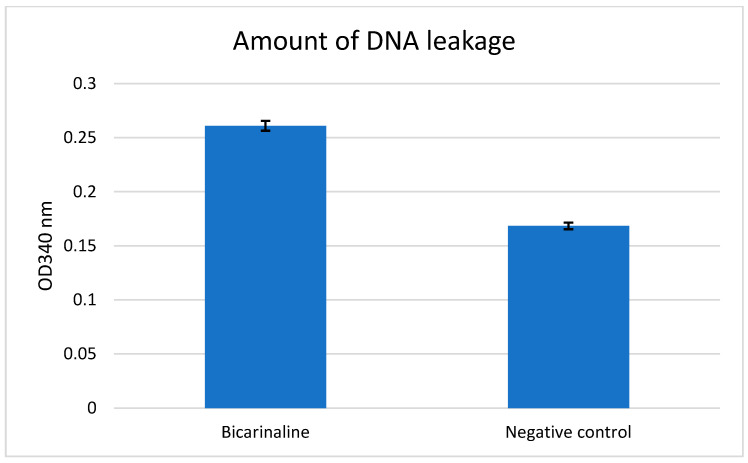
The comparison of DNA leakage between *H. pylori* and negative control when exposed to Bicarinalin at 340 nm. Bicarinalin shows better activity than negative control.

**Figure 4:**
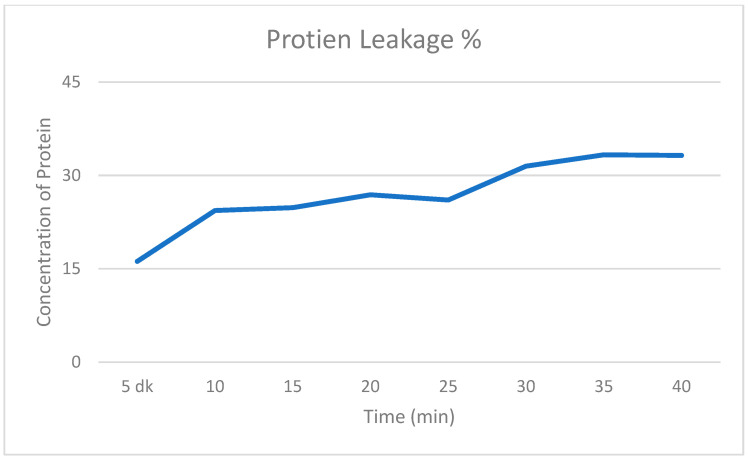
The graphic chart of protein leakage in percentage when *H. pylori* and negative control are exposed to Bicarinalin. Leakage increases with time.

The DNA and protein leakage assessment for *H. pylori* ATCC strain is shown in (Figure 3 and Figure 4).

According to the calculation results using the OD values specified in the method, protein leakage was found to have increased by 33.25% and DNA leakage by 55.10%. Bicarinalin has been demonstrated to cause greater leakage of cellular DNA and protein compared to the negative control.

### 2.5. Scanning Electron Microscope Analysis

**Figure 5:**
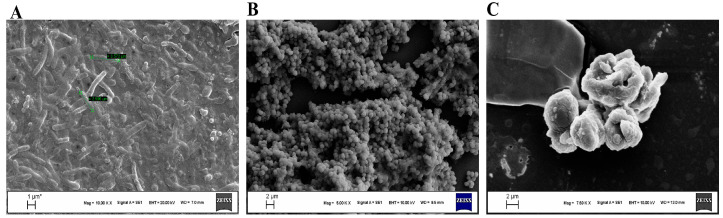
Scanning electron microscope (SEM) analysis of Bicarinalin effect on *H. pylori* cells. (**A**) *H. pylori* directly from biopsy tissue samples, without any effect. (**B**) *H. pylori* after culturing in the lab; the bacterial cells have transformed from spiral to coccoid. (**C**) *H. pylori* after treatment with Bicarinalin; the bacterial membranes have ruptured, and the cells have aggregated.

The morphological features and the cellular damage by Bicarinalin were confirmed by scanning electron microscope (SEM) analysis, which is demonstrated in (Figure 5). (Figure 5A) displays *H. pylori* ATCC cells from stomach tissue directly after the collection of the biopsy samples without using any antibiotic or chemicals. The cells are intact, spiral-rod shaped. (Figure 5B) displays the bacterial cells after culturing and incubation in MHB in the lab; the cells are transformed to coccoid shape. (Figure 5C) displays *H. pylori* cells after exposure to Bicarinalin; the cell membrane is ruptured, and the cells are shrunk and aggregated, and there are blebs on the outer membrane resulting in deformation and destruction of the bacterial cell membrane.

## 3. Discussion

In recent studies, researchers focus on finding and developing novel compounds with antimicrobial properties, either from natural or synthetic resources to overcome multidrug-resistant microorganisms [24]. Currently, antimicrobial peptides have attracted attention due to their diversity and potential as antimicrobial agents against resistant pathogens such as *H. pylori* which cause various disorders in humans [25]. Former studies have validated the antimicrobial effect of Bicarinalin derived from ant venom on a variety of pathogens including *H. pylori*. Based on this idea, we studied the activity and synergetic effect of Bicarinalin on *H. pylori*. The antimicrobial activity of Bicarinalin at predetermined concentrations was evaluated against the commercial reference strain of *H. pylori*. The results of the evaluation of the well diffusion assay revealed a dose-dependent increase in activity, accompanied by a corresponding enlargement of the inhibition zone diameter. A review of the literature reveals an intensive search for potential antibacterial agents targeting *H. pylori*. Among these, peptides such as AMPs, along with both derived and non-derived biomolecules, have attracted particular attention owing to their natural origin and low cytotoxicity potential [26,27]. In the study conducted by Nuding et al. [26], the activities of gastric AMPs HBD3, LL37, HBD1 and elafin were evaluated against *H. pylori* strains obtained from biopsies using the well diffusion method. It was observed that HBD3 (8–10 mm) and LL37 (12–15 mm) peptides formed zones in all test strains, while the expression of other AMPs did not increase during infection, and *H. pylori* showed resistance to some of them. It was observed that a concentration of 20 μg resulted in the formation of an effective zone diameter in susceptible strains [26]. In the study conducted by Moghaddam et al. [28], the activities of C11 peptide against *H. pylori* were evaluated in its pure form, encapsulated with chitosan and with added nanoparticles, and C11 showed an MIC value of 16 ug/mL in its pure form [28]. The performance of Bicarinalin against *H. pylori* is comparable to that of other antimicrobial peptides described in the literature. In particular, the zone diameters obtained at a concentration of 32 µg/mL (16.4–18.3 mm) are comparable to the effects recorded at similar concentrations for agents such as the CM11 peptide or the PHI derivative molecule.

This study demonstrates that Bicarinalin exhibits significant antimicrobial activity against commercial *H. pylori* ATCC strain. The MIC_99_ required to achieve 99% bacterial growth inhibition, reflecting consistent antibacterial potential. In particular, at high concentrations (15 μg/mL and 30 μg/mL), Bicarinalin exhibits stronger antimicrobial activity than standard antibiotics such as levofloxacin and amoxicillin, demonstrating significant therapeutic potential in clinical cases where antibiotic resistance is widespread. An examination of the activities of Bicarinalin against various pathogens reveals that its efficacy is dependent on the concentration employed. In a study conducted by Téné et al. [29], Bicarinalin was reported to be effective against different *Enterobacteriaceae* strains at a minimum MIC_50_ value of 12.8 μg·mL^−1^ using the microdilution method. In a study investigating the antibacterial effect of Bicarinalin, BP100, and Colistin AMPs against *Acinetobacter baumannii*, the MIC value of Bicarinalin was determined to be 4 μg/mL, the MIC value of Colistin was determined to be 0.5 μg/mL, and the MIC value of BP100 was determined to be 4 μg/mL using the microdilution technique [20]. In a similar study carried out by Guzman et al. [25], Bicarinalin was effective against *H. pylori* ATCC strain and clinical isolates with the MIC_50_ of 8.6 μg·mL^−1^ and 2.2 μg·mL^−1^, respectively. In addition, electron microscopy analysis showed that the compound significantly reduces the potential of *H. pylori* to adhere to gastric cells at the concentration of 0.25 μg·mL^−1^, but visible effect was not observed on the plasma membrane until the concentration was increased to 10 μg·mL^−1^. In our study, Bicarinalin exhibited effectiveness against *H. pylori* ATCC strain with the MIC_99_ of 4.8 μg/mL, recommending better activity than AMX, LEV, TET, and CLR to overcome *H. pylori* infection.

AMPs and synergistic combinations of antibiotics used in treatment are a promising strategy for increasing antibacterial efficacy, reducing effective antibiotic doses, and combating the development of resistance. This is achieved through mechanisms such as AMPs increasing membrane permeability, biofilm disruption, and direct enhancement of antibiotic activity [30]. A study reported that Magainin 2 and PGLa AMPs, which are effective against Gram-negative and Gram-positive bacteria and fungi when used individually, are more effective when used in combination [31]. As reported by Naghmouchi et al. [32], the antimicrobial peptide nisin Z, which alone shows no activity against Gram-negative bacteria, demonstrated synergistic effects against *Pseudomonas fluorescens* when combined with ampicillin [32]. Similarly, in the present study, Bicarinalin was found to be effective against *H. pylori* strains as a single agent, and its combination with LEV, CLR, and TOB resulted in a marked synergistic enhancement of antibacterial activity. The diameters of inhibition zones were recorded as follows: 14.2 mm for LEV, 8.9 for TOB, 7.3 for CLR. While Bicarinalin had a diameter of inhibition zone of 11 mm at the concentration of 15 μg/mL and 30 μg/mL and 12 mm for 45 μg/mL. Whereas the diameter of inhibition zones for Bicarinalin when combined with LEV was 20 mm, and for Bicarinalin when combined with CLR was 16 mm and similarly, 16 mm when Bicarinalin was combined with TOB. These results indicate that Bicarinalin increases the potency of the antibiotics when combined with them rather than when used alone, particularly LEV, suggesting that Bicarinalin has the potential to be used as novel agent against *H. pylori* infections.

The bacterial cytoplasmic membrane is essential for processes such as nutrient transport, osmoregulation, respiration, cell wall, and lipid synthesis. Maintaining its integrity is crucial, as its disruption can disrupt cellular homeostasis, impair metabolism, and lead to cell death. AMPs typically exert their bactericidal effects by targeting and disrupting this membrane, causing rapid permeability changes and irreversible structural damage, which ultimately leads to cell lysis [25,33]. In the study by Narayana et al. [34], the effectiveness of Epi-1 on *H. pylori* morphology was investigated using transmission electron microscope (TEM). Exposure to Epi-1 led to total membrane lysis, causing the release of cellular contents and eventual cell death. There were membrane blebs implying the creation of saddle-splay membrane curvature [34]. Tene et al. [29] conducted a membrane permeabilization assay to evaluate the effect of Bicarinalin on bacterial membranes. Using SYTOX Green, a membrane-impermeable DNA-binding fluorescent dye, they assessed the impact of Bicarinalin on live bacterial cells. An increase in fluorescence intensity following the addition of Bicarinalin at varying concentrations to *Enterobacter* or *Cronobacter* suspensions indicated membrane disruption. Similarly, in our study the SEM analysis confirmed that exposure to Bicarinalin resulted in the shrinkage and aggregation of the *H. pylori* cells, the rupture of the membrane, and blebs were seen on the surface of the membranes. Similarly in the same study by Eales et al. [20], SEM analysis revealed that there are more morphological alterations as the doses of the agents rise. The changes include blebbing, more variable shape and shrinkage of the cells, and disruption of the membrane. In addition, the surface coverage decreases as well. These results indicate that Bicarinalin uses the same mechanism of action on different microorganisms.

The Bradford method for estimating the leakage of protein and using colorimetric assay for detecting DNA is an attractive method to show the potential of compounds to affect the pathogens on a cellular level. This approach has been demonstrated by Han et al. [35], that detected the leakage of cellular protein and DNA of *H. pylori* through colorimetric assay using spectrophotometer. Nisin was combined with lactic acid which resulted in leakage of protein and DNA contents of the bacteria. A similar effect was exhibited by Ampicillin. However, there was no considerable effect when nisin and lactic acid were used alone. In contrast, the data in our study exhibited that Bicarinalin alone increases the leakage of DNA by 55.10% and protein by 33.25%, demonstrating the ability of Bicarinalin to damage the membrane of *H. pylori* and pass through the cellular membrane, resulting in damage to the cellular structure and leading to the leakage of protein and DNA to the external environment.

The most significant challenge to the clinical utilization of AMPs is the substantial diminution in their efficacy in saline environments and physiological conditions, such as serum. Valdivieso-Rivera and colleagues have demonstrated that MIC values increase threefold under these conditions [36]. In addressing this issue, Mendes et al. demonstrated that lipid modification and chain shortening of peptides increased their activity and preserved their antibacterial, antibiofilm, and membranolytic activities in physiological conditions containing serum and salt. It is hypothesized that the optimization of synthetic peptides could circumvent the limitations of natural peptides and yield potent antimicrobials with greater clinical relevance [37].

## 4. Materials and Methods

### 4.1. The Synthesis of Bicarinalin

The C-terminal amidated peptide, Bicarinalin with the sequence of (KIKIPWGKVKDFLVGGMKAV-NH2), has been synthesized using a Liberty microwave-assisted automated peptide synthesizer (Genscript, Piscataway, NJ, USA) with 96% purity degree. As formerly defined in [29].

### 4.2. Bacterial Strains

The reference (ATCC 43504) has been used in this study.

### 4.3. Bacterial Isolation and Identification

The bacterial suspensions were restored through growing in a Brucella broth as previously described by Whitmire and Merrell [38], with the addition of 500 µL Fetal Bovine Serum (FBS) and 5 µL Vancomycin, the tubes were placed in an incubator at 37 °C with shaking at 100 rpm for 5–7 days. A microaerophilic condition (5% O_2_ and 10% of CO_2_) was developed using the Anaerocult™ (Oxoid) anaerobic jar system. After incubation, the suspensions were transferred from brucella broth and streaked onto CBA plates enriched with 7% horse blood and incubated at 37 °C under a condition of 5% O_2_ and 10% CO_2_ for 72 h. Using the Anaerocult™ (Oxoid) anaerobic jar system. Small colonies were grown, and *H. pylori* strains were identified microscopically by Gram-staining technique and biochemical tests (catalase, oxidase, and positive urease) [39]. Urease test was performed to indicate the presence of Urease. The characterized *H. pylori* strains were resuspended in 7 mL of Brain Heart Infusion Broth (BHIB) supplemented with 1 mL FBS and 2 mL Glycerol according to Whitmire and Merrell [38]. The mixture was stored at −20 °C for future analysis.

### 4.4. Well Diffusion

The activity of the antibiotics (AMX, TET, CLR, LEV, TOB) and Bicarinalin against the referenced strain of *H. pylori* (ATCC 43504) was evaluated through well diffusion techniques. Bacterial suspensions from BHIB were thawed and were adjusted to 2 McFarland standard and streaked onto MHA supplemented with horse blood. Different concentrations of Bicarinalin with serial dilution were prepared and placed into the wells on MHA. The antibiotic disks were placed into the wells separately. The plates were incubated at 37 °C under a condition of 5% O_2_ and 10% CO_2_ for 72 h. After incubation, the diameters of inhibition zones on the culture media were measured in millimeters and recorded as the lowest concentration where Bicarinalin and the antibiotics completely inhibited visible growth in the culture.

### 4.5. Microdilution Assay

Minimal inhibitory concentration (MIC_99_) of Bicarinalin was indicated through a standard broth microdilution assay. An amount of 3 mg of Bicarinalin was dissolved in 1 mL sterile water, and different concentrations were prepared with serial dilution, including 15 µg/mL, 30 µg/mL, and 45 µg/mL. Bacterial suspensions of *H. pylori* from the referenced strain of *H. pylori* (ATCC 43504) were adjusted to 2 McFarland standard and placed into 96-well microplates with 280 µL Muller Hinton Broth medium. Bicarinalin from each concentration was added to the wells, bacteria alone in the wells without Bicarinalin was used as the negative control, bacteria with LEV and AMX was used as positive control. The microplate incubated in Panasonic (MCO-18ACL-PA) CytoGrow CO_2_ incubator at 37 °C in a condition of 5% O_2_ and 10% CO_2_ for 48 h. The MIC_99_ values were obtained through measuring the optical density (OD) by scanning the culture plates in the tunable microplate reader (VERSAmax), USA at 600 nm wavelength. The MIC_99_ was reported as the lowest concentration of Bicarinalin at which there was no visible growth. The concentration of the substance at which growth was inhibited by 99% (MIC_99_) was calculated using the following formula Erdoğan Eliuz [40]. Each experiment was conducted in triplicate, with the resulting measurements averaged three times.

$$\mathrm{Inhibition}\;(\%)=\left[\frac{1-OD\;test\;well}{OD-Corresponding\;control\;well}\right]\;\times \;100$$

### 4.6. Synergistic Activity of Bicarinalin

The synergistic activity of Bicarinalin in combination with the antibiotics (LEV, CLR, and TOB) against the reference strain of *H. pylori* (ATCC 43504) was determined using well diffusion method. The bacteria were streaked on MHA supplemented with sheep blood, and 12.5 µL of Bicarinalin from the 30 µg/mL concentration was mixed with each antibiotic and placed on the wells on the plate. Bicarinalin and each antibiotic were placed separately on other plates as control. The plates incubated in Panasonic (MCO-18ACL-PA) CytoGrow CO_2_ incubator at 37 °C under a condition of 5% O_2_ and 10% CO_2_ for 48 h. The diameters of inhibition zones in the culture media were measured in millimeters and were recorded as the lowest concentration where Bicarinalin in combination with the antibiotics completely inhibited growth in the culture.

### 4.7. Assessment of Intracellular Protein and DNA Leakage

Protein and DNA leakage was assessed according to the technique described by Erdoğan Eliuz [40]. Bacterial suspensions from the reference strain *H. pylori* (ATCC 43504) were adjusted to 0.5 McFarland using PBS and were incubated with Bicarinalin from the 45 µg/mL concentration in Panasonic (MCO-18ACL-PA) CytoGrow CO_2_ incubator at 37 °C under a condition of 5% O_2_ and 10% CO_2_ for 3 h. Then the mixture was centrifugated at 14,000× *g* for 3 min. An amount of 50 µL from the supernatant was placed into the 96-well plate and dyed with 50 µL from Bradford dye reagent for 5 min for the development of color change. PBS with Bradford reagent dye were used as the negative control. The absorbance was measured every 5 mins for 8 times at 595 nm wavelength using tunable microplate reader (VERSAmax, Ocean Springs, MS, USA). For DNA leakage assessment, 50 µL supernatant of the mixture was withdrawn into the 96-well plate and PBS was used as the negative control. The absorbance was measured at 340 nm wavelength through the same microplate reader. The experiment was repeated three times.

The increase in the leakage of DNA and protein is expressed as

$$\mathrm{DNA}\;\mathrm{or}\;\mathrm{Protein}\%=\left[\frac{{OD}_{x}-{OD}_{c}}{{OD}_{c}}\right]\;\times \;100$$

OD_x_ represents absorbance of treated samples, whereas OD_c_ represents absorbance of control samples.

### 4.8. SEM Analysis

Bicarinalin from the concentration of 128 μg/mL was mixed with bacterial suspensions from the referenced strain of *H. pylori* (ATCC 43504) in MHB and incubated at 37 °C under a condition of 5% O_2_ and 10% CO_2_ using the Anaerocult™ (Oxoid) anaerobic jar system for 48 hrs. The mixture was centrifuged for 5 mins at 5000 rpm and then pallet of the microorganisms was collected. The cells were washed two times with PBS and the supernatant was removed. Then the cells were fixed with 3% glutaraldehyde and placed in the fridge at 4 °C for 2.5 h. Then the cells were centrifugated and the supernatant was removed, and Ethanol was added with different concentrations as follows: 30–50–70–90–100% (after each addition, the cells were centrifugated and the supernatant was removed). Then the cells were left to dry at room temperature.

The morphological features of the samples were characterized using a ZEISS EVO50 brand-model scanning electron microscope (ZEISS EVO50 SEM, Oberkochen, Germany) on a secondary electron (SE) detector, at an acceleration voltage of 15 kV, at approximately 8–11 working distance (WD). After the test samples were fixed in plastic Petri dishes, the materials were cut smoothly with the help of a suitable cutter. Then, for electron microscope imaging, the samples were made conductive at a thickness of 5 nm in a gold coating device (electron microscope system). In the test samples, images were taken from the areas where bacteria adhered most densely. Analyses were made at Harran University, Science and Technology Application and Research Center (HUBTAM).

### 4.9. Statistical Analysis

Statistical data analyzes were performed using one-way ANOVA with post hoc Turkey HSD Test.

## 5. Conclusions

In conclusion, the present study demonstrates that Bicarinalin exhibits inhibitory activity against *H. pylori* and has the potential to enhance the antibacterial effects of certain antibiotics. While these results are encouraging, they are insufficient to prove a definitive synergistic interaction. Conversely, the findings of this study suggest a potential enhancing effect that merits further investigation. It is recommended that future studies encompass in vivo validation through the implementation of more rigorous methodologies. Such methodologies may include time–kill kinetics and fractional inhibitory concentration index analyses. These studies will contribute to a more comprehensive elucidation of Bicarinalin’s therapeutic potential, pharmacokinetics, and safety profile in combination therapy.

## Figures and Tables

**Table 1:** Diameters of inhibition zones of Bicarinalin (μg/mL) through well diffusion method.

Concentration of Bicarinalin	4 μg/mL	8 μg/mL	16 μg/mL	32 μg/mL
Inhibition zone	7.8 mm	8.7 mm	9.7 mm	18.3 mm

**Table 2:** The inhibition zones (in mm) of the antibiotics of Bicarinalin with different concentrations alone and when combined with the antibiotics against *H. pylori* through well diffusion method MHA.

Antibiotics/Bicarinalin	Inhibition Zones (in mm)
LEV	14.2 mm
CLR	7.3 mm
15 μg/mL	11 mm
30 μg/mL	11 mm
45 μg/mL	12 mm
LEV + Bic	20 mm
CLR + Bic	16 mm

## Data Availability

The data presented in this study are available on request from the corresponding author. The data are not publicly available due to institutional restrictions.
